# Genetic Association of *PPARGC1A* Gene Single Nucleotide Polymorphism with Milk Production Traits in Italian Mediterranean Buffalo

**DOI:** 10.1155/2021/3653157

**Published:** 2021-03-18

**Authors:** Seyed Mahdi Hosseini, Ye Tingzhu, Majid Pasandideh, Aixin Liang, Guohua Hua, Farmanullah, Nicola M. Schreurs, Sayed Haidar Abbas Raza, Angela Salzano, Giuseppe Campanile, Bianca Gasparrini, Liguo Yang

**Affiliations:** ^1^Key Laboratory of Animal Genetics, Breeding and Reproduction, Ministry of Education, College of Animal Science and Technology, Huazhong Agricultural University, Wuhan, Hubei 430070, China; ^2^Department of Animal Science, Sari Agricultural Sciences and Natural Resources University, Sari, Mazandaran, Iran; ^3^Faculty of Veterinary and Animal Sciences, National Center for Livestock Breeding Genetics and Genomics LUAWMS, Uthal, Balochistan, Pakistan; ^4^Animal Science, School of Agriculture and Environment, Massey University, Palmerston North, New Zealand; ^5^College of Animal Science and Technology, Northwest A&F University, Yangling, Shaanxi 712100, China; ^6^Department of Veterinary Medicine and Animal Production, Federico II University, Naples, Italy

## Abstract

*PPARGC1A* gene plays an important role in the activation of various important hormone receptors and transcriptional factors involved in the regulation of adaptive thermogenesis, gluconeogenesis, fiber-type switching in skeletal muscle, mitochondrial biogenesis, and adipogenesis, regulating the reproduction and proposed as a candidate gene for milk-related traits in cattle. This study identified polymorphisms in the *PPARGC1A* gene in Italian Mediterranean buffaloes and their associations to milk production and quality traits (lactation length, peak milk yield, fat and protein yield, and percentage). As a result, a total of seven SNPs (g.-78A>G, g.224651G>C, g.286986G>A, g.304050G>A, g.325647G>A, g.325817T>C, and g.325997G>A) were identified by DNA pooled sequencing. Analysis of productivity traits within the genotyped animals revealed that the g.286986G>A located at intron 4 was associated with milk production traits, but the g.325817T>C had no association with milk production. Polymorphisms in g.-78A>G was associated with peak milk yield and milk yield, while g.304050G>A and g.325997 G>A were associated with both milk yield and protein percentage. Our findings suggest that polymorphisms in the buffalo *PPARGC1A* gene are associated with milk production traits and can be used as a candidate gene for milk traits and marker-assisted selection in the buffalo breeding program.

## 1. Introduction

Water buffalo provides 5% of the world's milk supply, and their milk is high in protein, fat, lactose, and minerals compared to cow's milk. The genome is organized in five pairs of metacentric chromosomes, 19 pairs of acrocentric chromosomes, and two sex chromosomes, X and Y. There are two types of domestic water buffalo, the riverine buffalo (*Bubalus bubalis*, 2*n* = 50) and the swamp buffalo (*Bubalus carabanesis*, 2*n* = 48 [1, 2]. They differ significantly in milk yield, approximately 2200-2400 kg/year for the riverine buffalo and 500-700 kg/year for the swamp buffalo [3]. European buffalo is all of the river types, and in Italy, the Mediterranean type is selected from other European breeds but differs genetically [4]. Mediterranean buffalo in Italy produces more than 2,000 kg of milk in 270-day lactation with 8.1% fat and 4.6% protein (Associazione Nazionale Specie Bufalina, http://www.anasb.it). Genome sequencing and *de novo* assembly for the Italian Mediterranean buffalo were done using the MaSuRCA assembler [5] by the International Water Buffalo Genome Consortium (GCF_000471725.1; deposited on NCBI in November 2013) [18]. Detection of genes that influence important production traits is an essential field of research in livestock species. Recent studies revealed some candidate genes harboring single nucleotide polymorphisms (SNP) significantly associated with the fatty acid composition [6, 7], protein percentage [8, 9], and milk fat content [10, 11] on different buffalo chromosomes. Also, some polymorphisms associated with milk yield (kilograms of milk per lactation) have been identified at the whole buffalo genome level [12–16]. These studies have made great contributions to the progress of various breeding programs in buffalo.

The PGC-1 family consists of three members, namely, *PPARGC1A* (*PGC-1α*), *PPARGC1B* (*PGC-1β*), and PGC-related coactivator [17]. Peroxisome proliferator-activated receptor-*γ* coactivator-1*α* (*PPARGC1A*) is located in the middle of chromosome 7 of river buffalo (BBU7) (Goldammer et al. 2007) and chromosome 6 in cattle (BTA6) [18]. Buffalo chromosome 7 is the homolog of cattle chromosome 6 known for its association with milk production traits [19, 20]. The *PPARGC1A* gene for both river and swamp buffalo is all composed of 797 amino acid residues. The molecular weight of the buffalo *PPARGC1A* gene is about 90.49 kDa. Secondary structure analysis of buffalo *PPARGC1A* gene consists of 31.37% *α*-helix, 4.27% *β* turn, 11.67% extended strand, and 52.70% random coils [21]. *PPARGC1A* gene plays an important role in the activation of various important hormone receptors and transcriptional factors involved in the regulation of adaptive thermogenesis, fiber-type switching in skeletal muscle, mitochondrial biogenesis, adipogenesis, and gluconeogenesis [17, 22, 23]. It regulates processes affecting reproduction [24, 25]. The *PPARGC1A* gene in cattle was proposed as a candidate gene for milk-related traits ([18, 26, 27]; Pasandideh, et al., 2015; [28]) and in buffalo for milk fat [21]. Polymorphism of *PPARGC1A* gene in Czech Fleckvieh cattle was associated with milk yield, milk fat, and protein yield and in particular, the T allele positively associated with both milk fat percentage [29]. In Italian Holstein cattle, the SNPs of the *PPARGC1A* gene located in BTA6 were associated with protein yield, protein percentage, and milk yield [26]. So far, multiple polymorphic sites have been found in the cattle *PPARGC1A* gene. In dairy cows, there was a significant association between c.1790+514G>A, c.1892+19T>C, and c.1892+19G>A of *PPAGC1A* gene and milk fat yield [20, 28]. However, there are few reports about the SNPs of the *PPARGC1A* gene in buffalo. [21] found eight SNPs in river and swamp buffalo and showed that buffalo *PPARGC1A* gene is an inducible transcriptional coactivator involved in regulating carbohydrate and fat metabolism in buffalo and may participate in milk fat synthesis.

Due to previous association studies with milk production traits in dairy cattle, we hypothesized that the *PPARGC1A* gene is associated with milk production traits in buffalo. However, there is no enough examined research, so this study was performed for investigating the relationship between *PPARGC1A* gene polymorphisms and different milk traits in dairy Mediterranean buffalo. These results can provide useful information about the development of breeding programs for the Mediterranean buffalo and dairy buffalo industry.

## 2. Material and Methods

### 2.1. Ethics Statement

The data in this study were obtained by the Italian Buffalo Breeders' Association (ANASB). The animal treatment protocols and experimental design were approved by The Ethical Animal Care and Use Committee of the University of Naples “Federico II.”

### 2.2. Animal Resources and Phenotypic Data

A total of 855 lactation records were collected from 346 Italian Mediterranean buffaloes born from 2004 to 2014 with 1–7 parities and located in 4 experimental herds in southern Italy. Seven milk production traits including lactation length (LL), peak milk yield (PM), total milk yield (MY), fat yield (FY), fat percentage (FP), protein yield (PY), and protein percentage (PP) were recorded. All of the milk production records were adjusted to a 270-day lactation length [30, 31].

### 2.3. DNA Extraction from Blood

Buffalo genomic DNA was extracted from whole-blood samples using the QIAamp DNA Blood kit (Qiagen, Milano, Italy). The purity and concentration of DNA were measured by spectrophotometry using the NanoDrop 2000 analyzer (Thermo Fisher Scientific, Wilmington).

### 2.4. Primer Design, Sequencing, and Genotyping

The whole gene sequence of the *PPARGC1A* gene in *Bubalus bubalis* (river buffalo) was downloaded from NCBI (https://www.ncbi.nlm.nih.gov/, GenBank accession number: XM_025290240.1). The SNP primers were designed by Primer Premier 5.0 software (Premier Biosoft, Palo Alto, CA) and synthesized by TSINGKE Biological Technology (Beijing, China). To identify SNPs in the promoter of this gene, a sequence of 2000 bp before it was also downloaded, which we usually regarded as a promoter of a gene. The identification of the SNP was performed by sequencing. Twenty-five pairs of primers were designed and amplified at the 5′ upstream region, 3′ UTR, 3′ downstream region, exonic, and adjacent intronic regions of the buffalo PPARGC1A gene. This was performed using 25 *μ*l of 50 ng genomic DNA, 12.5 *μ*l 2x reaction mix (including 500 *μ*M dNTP each; 20 mM Tris–HCl; pH 9; 100 mM KCl; 3 mM MgCl_2_ and 0.5 units of Taq DNA polymerase) and 0.5 *μ*M of each primer. The cycling protocol was as follows: 5 min at 95°C, 35 cycles of denaturing at 95°C for 30 s, annealing at Tm°C for 30 s, and extension at 72°C for 30 s, with a final extension at 72°C for 10 min. PCR products were visualized by electrophoresis on 2% agarose gel using 1x AE buffer and sent for sequencing to the company (Sangon Biotech, Wuhan, China). Mutations were identified from the aligned sequencing results using the Chromas software (https://technelysium.com.au/wp/chromas/). The SNPs were genotyped by the company (Compass Biotechnology, Beijing, China) using the iPLEX MassARRAY SNP searching method.

### 2.5. Statistical Analysis

The Pop Gene software (Version 1.31, 1999) was used to estimate the Hardy-Weinberg test (*P* value ≤ 0·05), gene, and genotype frequency. The effects of *PPARGC1A* gene genotypes on milk production traits were analyzed using the least squares method of the GLM procedure of SAS (Statistical Analysis System, Version 9.2). The Tukey honest significant difference method was used for multiple comparisons between different genotypes. The used model was as follows:

(1) $$\begin{array}{c} y_{ijkl}=\mu +{\text{S}}_{\text{i}}+{\text{P}}_{\text{j}}+{\text{G}}_{\text{k}}+{\text{SNP}}_{l}+{\text{e}}_{\text{ijkl}}, \end{array}$$

where *y*_*ijkl*_ is the observation of milk production traits, *μ* is the overall mean, *S*_*i*_ is the random effect of sire, *P*_*j*_ is the fixed effect of the parity, *G*_*k*_ is the fixed effect of contemporary group constructed with the effects of season-herd and year, SNP_*l*_ is the fixed effect of the genotypes, and *e*_*ijklm*_ is the residual effect of each observation.

The percentage of the additive genetic variance justified by the significant SNP was estimated as follows [32]:

(2) $$\begin{array}{c} \hat{a}=\frac{\text{BB}-\text{AA}}{2},\\ \hat{d}=\text{AB}-\frac{\text{BB}+\text{AA}}{2},\\ \alpha \text{i}=\hat{a}+\left(q-p\right)\hat{d},\\ \%V_{i}=100\cdot \frac{2p_{i}q_{i}{\alpha_{i}}^{2}}{\sigma_{g}^{2}}, \end{array}$$

where *p*_*i*_ and *q*_*i*_ are the allele frequencies, $\hat{a}$ is the additive and $\hat{d}$ is the dominance effects for genotypes (AA, AB, and BB), *α*_*i*_ is the allele substitution effect, and *σ*_*g*_^2^ is the genetic variance estimated for the trait by WOMBAT software [33].

## 3. Results and Discussion

### 3.1. Characterization of *PPARGC1A* SNPs and Genotype Allelic Frequencies

A total of 7 SNPs were detected in the *PPARGC1A* gene of which one was located in the promotor, 4 in exons, and 2 in introns of chromosome 7 in Italian Mediterranean buffalo (Table 1). All 7 SNPs were genotyped. The allelic and genotypic frequencies, observed and expected heterozygosity, and the value of the chi-squared test (*χ*^2^) are shown in Table 2. The results of *χ*^2^ showed that the genotypic frequencies of the polymorphisms were within the Hardy-Weinberg equilibrium (*P* < 0.05), indicating the population has been under selection for milk production and composition traits. In the examined group of 346 buffaloes, the genotypes were 160 AA, 128 GA, and 24 GG at g.-78A>G; 49 CC, 155 CG, and 143 GG at g.224651G>C; 30 AA, 146 GA, and 168 GG at g.286986G>A; 92 AA, 163 GA, and 89 GG at g.304050G>A; 92 AA, 167 AG, and 89 GG at g.325647G>A; 76 CC, 119 TC, and 67 TT at g.325817T>C, and 92 AA, 167 AG, and 89 GG at g.325997G>A. In this study, the highest and lowest genotypic and allele frequencies were reported for AA (0.51) and GG (0.08) genotypes and A (0.72) and G (0.28) alleles in SNP1 (g.-78A>G), respectively. The differences in genotypic frequencies might be regarded as the reason for the variation of the studied breeds or discrepancy in sample size. One study [34] reported an SNP located at the c.1599A>T on the exon 8 of *the PPARGC1A* gene in Anatolian water buffaloes. In this population, the frequencies of A and T alleles were estimated at 0.768 and 0.232, respectively, and heterozygosities were calculated 24.59% in this population lower than our results. The highest frequency in our study was A (0.72) that was close to this research. Additionally, they showed disagreement with the expected Hardy-Weinberg balance by chi-squared (*χ*^2^) analyses (*P* < 0.001) that were not in agreement with our result. [35, 36] identified six buffalo-specific SNPs in the *PPARGC1A* gene (positions C718T in intron 3, A1844G in exon 6, C1902T in intron 6, and G2382T, C2529T, and A2657G in exon 8) in Indian buffaloes. Two SNPs were nonsynonymous, resulting in amino acid changes at positions W346G and F395L. Additionally, [35, 36] identified 2 loci for these SNPs (*PPARGC1A_Eco0109I* and *PPARGC1A_AciI*) located in exon 8 in Indian buffaloes. As a result, it is concluded that exon 8 is a common location for identified SNPs in buffaloes.

### 3.2. Analysis of *PPARGC1A* SNPs with 7 Milk Production Traits

Table 3 shows the effects of 7 identified SNP position genotypes of the *PPARGC1A* gene on milk production traits in Italian Mediterranean buffalo. In total, the significant SNPs were justified as 1.84, 0.93, and 0.82 of total genetic variance for MY, PP, and PM traits, respectively, whereas one significant SNP for FY, FP, and PY accounted for 0.35, 0.25, and 0.17 of the total genetic variance. In this study, no differences were found between the polymorphisms of the *PPARGC1A* gene in buffalo for lactation length. This result was in agreement with [34] who reported an effect on the initiation and maintenance of lactation length in Anatolian water buffaloes. The analysis revealed SNP1 and SNP3 to be associated with peak milk yield (*P* < 0.05). In both of these SNPs, the homozygote GG animals were found to have greater (*P* < 0.01) peak milk yield than heterozygote GA and homozygote AA. The SNP1, SNP3, SNP4, SNP5, and SNP7 with genotypes GG, GA, and AA are associated (*P* < 0.05) with 270 d milk yield and the polymorphisms of 4 positions (SNP3, SNP4, SNP5, and SNP7) with genotype GG having a greater milk yield than AG and AA genotypes whereas SNP1 with the heterozygous genotype GA had the greatest milk yield (*P* < 0.01). In Jersey cows, no difference in milk yield was observed between T>C and A>C genotypes [37]. However, in agreement with the current study, *PPARGC1A* gene polymorphisms were associated with differences in milk yield in Czech Fleckvieh cattle [29] and Italian Holstein cattle [26]. Similar to the genotypes of the Italian Mediterranean buffaloes studied, [18] reported that the A allele in the A>C position of *PPARGC1A* gene was associated with lower milk yield in the North American Holstein whereas they did not find any association with the T>C position and milk production traits which were also observed in an earlier study with German Holstein [28]. In the current study, the SNP3 genotypes of the *PPARGC1A* gene differences in fat yield and percentage were found with that of the GG genotype associated with greater fat yield compared to other genotypes (*P* < 0.01) whereas buffaloes of the AA genotype had a greater fat percentage compared to GA and GG buffaloes. *PPARGC1A* gene plays a fundamental role in lipid metabolism and is a candidate gene underlying the QTL variation for milk fat-related traits on bovine chromosome 6 [38]. However, [28] showed that the SNP T>C of the *PPARGC1A* gene did not affect milk fat percentage traits in German Holstein cattle. Likewise, no difference in milk fat percentage between SNPs T>C and A>C genotypes was reported by [37] in Jersey cows. These findings contradict the association between the SNP T>C and milk fat composition in Dutch Holstein-Friesian cattle [20], Czech Fleckvieh cattle [29], and Iranian Holstein cattle (Pasandideh et al., 2015). Additionally, (Pasandideh et al., 2015) reported that the *PPARGC1A* gene plays an essential role in lipid metabolism because it controls the high additive genetic variance of the milk fat.

So far, multiple polymorphic sites have been found in the cattle *PPARGC1A* gene. In dairy cows, there was a significant association between c.1790+514G>A, c.1892+19T>C, and c.1892+19G>A of *PPAGC1A* gene and milk fat yield [20, 28]. However, there are few reports about the SNPs of the *PPARGC1A* gene in buffalo. In agreement with our study, [21] found eight SNPs in buffalo. They indicated that the buffalo *PPARGC1A* gene is an inducible transcriptional coactivator involved in regulating carbohydrate and fat metabolism in buffalo and may participate in milk fat synthesis. Additionally, [34] found c.1598A>T polymorphism in exon 8 of *the PPARGC1A* gene. By determining the effects of c.1598A>T and other SNPs in the *PPARGC1A* gene on growth, they reported an effect on the initiation and maintenance of lactation, milk yield, quality of milk, and milk fat in Anatolian water buffaloes.

The SNP3 of the Italian Mediterranean buffaloes influenced protein yield (*P* < 0.05). Individuals with the homozygous genotype GG had a greater protein yield compared with homozygous genotypes AA (*P* < 0.01). Five polymorphisms (SNP1, SNP3, SNP4, SNP5, and SNP7) were associated (*P* < 0.05) with the protein percentage. The homozygous genotypes AA in SNP3 had a greater protein percentage than other genotypes (GA, GG, CC, and CG) (*P* < 0.01). [37] observed no significant difference between SNP T>C and A>C genotypes and protein percentage in Jersey cows. However, similar to the findings in the Italian Mediterranean buffaloes, [18] identified associations between SNP A>C of the *PPARGC1A* gene and milk protein percentage in the North American Holstein. Likewise, (Pasandideh et al., 2015) found an association between polymorphism T>C genotypes with milk protein yield in Iranian Holstein.

Different superscripts in the same row refer to significantly different at *P* < 0.01. *n*: number of animals; %Va: the percentage of the additive genetic variance justified by the significant SNPs.

## 4. Conclusions

Polymorphisms of the *PPARGC1A* gene were identified in the Italian Mediterranean buffalo. The polymorphisms of SNP1 showed a significant effect on peak milk yield and total lactation milk yield; SNP2 was associated with protein percentage, SNP3 with peak milk, milk yield, fat yield, fat percentage, protein yield, and protein percentage, and SNP4, SNP5, and SNP7 with milk yield and protein percentage. The SNP6 had no association with milk traits. This work could provide suggestions for molecular markers of milk production traits that could be used for selective breeding of Italian Mediterranean buffalo.

## Figures and Tables

**Table 1:** Primer sequences used for amplification of genomic sequence and identified SNPs for the *PPARGC1A* gene in Italian Mediterranean buffalo.

Primer set	Primer sequence	Length (bases)	PCR product size (bp)	Identified SNP	SNP location
PPARGC1AF_1_ PPARGC1AR_1_	5′-TGCTGTGTTGGGGGAAGATG-3′ 3′-GGGCTGAAAGAGATGAGAGAGTGA-5′	20 24	663	g.-78A>G	Promotor
PPARGC1AF_2_ PPARGC1AR_2_	5′-AGATCCCACATGCCACAACT-3′ 3′-GACAGCAAAGCAAGAACCCA-5′	20 20	780	g.224651G>C	Intron 1
PPARGC1AF_3_ PPARGC1AR_3_	5′-TTTCTCGCTTTCCCTCCTTC-3′ 3′-ACGCTCCGTTTTCACTCACA-5′	20 20	654	g.286986G>A	Intron 4
PPARGC1AF_4_ PPARGC1AR_4_	5′-AGTGGACACGAGGAAAGGAAG-3′ 3′-GGGTGGGTTTTGACAAGGTT-5′	21 20	724	g.304050G>A	Exon 8
PPARGC1AF_5_ PPARGC1AR_5_	5′-TGAACACATGCACCCCATCAT-3′ 3′-CGTGCCAGGAGTTTGGTTGT-5′	21 20	789	g.325647G>A g.325817T>C g.325997G>A	Exon 13 Exon 13 Exon 13

**Table 2:** Summary of population genetic information for 7 identified *PPARGC1A* SNPs in Italian Mediterranean buffalo.

SNP ID	Loci	Genotypic frequency			Allelic frequency		Heterozygosity		*χ* ^2^
							Observed	Expected	
SNP1	g.-78A>G	AA 0.51	GA 0.41	GG 0.08	A 0.72	G 0.28	0.41	0.40	0.01
SNP2	g.224651G>C	CC 0.14	CG 0.45	GG 0.41	C 0.36	G 0.64	0.44	0.46	0.68
SNP3	g.286986G>A	AA 0.10	GA 0.42	GG 0.48	A 0.30	G 0.70	0.43	0.42	0.04
SNP4	g.304050G>A	AA 0.27	GA 0.47	GG 0.26	A 0.51	G 0.49	0.48	0.50	0.79
SNP5	g.325647G>A	AA 0.26	GA 0.48	GG 0.25	A 0.51	G 0.49	0.48	0.50	0.45
SNP6	g.325817T>C	CC 0.29	TC 0.46	TT 0.25	C 0.52	T 0.48	0.45	0.50	0.05
SNP7	g.325997G>A	AA 0.27	GA 0.48	GG 0.25	A 0.51	G 0.49	0.48	0.50	0.45

**Table 3:** Effects of 7 identified SNP position genotypes of *PPARGC1A* gene on milk traits in Italian Mediterranean buffalo.

Polymorphism	Traits	AA (*n* = 160)	GA (*n* = 128)	GG (*n* = 24)	*P* value	%Va
g.-78 A>G	LL (d)	258.33 ± 8.31	261.11 ± 7.23	258.22 ± 10.02	0.4454	—
	PM (kg)	14.44^b^ ± 0.05	14.71^ab^ ± 0.04	15.15^a^ ± 0.06	0.0372	0.53
	MY (kg)	2782.60^b^ ± 173.87	2829.52^a^ ± 180.45	2784.48^b^ ± 161.12	0.0399	0.41
	FY (kg)	222.02 ± 7.12	226.14 ± 6.14	219.07 ± 8.18	0.0731	—
	FP (%)	8.00 ± 0.26	8.02 ± 0.28	7.89 ± 0.19	0.5061	—
	PY (kg)	128.46 ± 5.55	130.40 ± 6.61	129.06 ± 5.14	0.0568	—
	PP (%)	4.63 ± 0.13	4.62 ± 0.15	4.67 ± 0.19	0.1206	—
		CC (*n* = 49)	CG (*n* = 155)	GG (*n* = 143)		
g.224651 G>C	LL (d)	259.46 ± 10.12	260.15 ± 9.55	258.80 ± 8.32	0.6352	—
	PM (kg)	14.65 ± 0.08	14.45 ± 0.05	14.59 ± 0.08	0.8793	—
	MY (kg)	2854.38 ± 180.32	2782.36 ± 180.49	2784.21 ± 178.36	0.7897	—
	FY (kg)	226.31 ± 8.02	223.77 ± 7.90	220.09 ± 8.65	0.4439	—
	FP (%)	7.97 ± 0.18	8.07 ± 0.22	7.94 ± 0.26	0.2952	—
	PY (kg)	128.10 ± 4.90	128.65 ± 4.88	129.12 ± 4.67	0.7486	—
	PP (%)	4.55^b^ ± 0.15	4.63^a^ ± 0.13	4.64^a^ ± 0.16	0.0012	0.21
		AA (*n* = 30)	GA (*n* = 46)	CG (*n* = 168)		
g.286986 G>A	LL (d)	260.31 ± 7.23	258.76 ± 11.12	259.76 ± 9.67	0.6976	—
	PM (kg)	13.58^b^ ± 0.06	14.54^a^ ± 0.06	14.70^a^ ± 0.05	0.0014	0.29
	MY (kg)	2596.17^b^ ± 190.55	2754.06^a^ ± 189.82	2857.58^a^ ± 185.94	0.0425	0.12
	FY (kg)	210.00^b^ ± 8.38	217.75^b^ ± 8.92	228.55^a^ ± 8.73	0.0001	0.35
	FP (%)	8.15^a^ ± 0.14	7.92^b^ ± 0.12	8.04^ab^ ± 0.11	0.0420	0.25
	PY (kg)	122.68^b^ ± 3.73	127.10^ab^ ± 4.74	131.09^a^ ± 4.90	0.0024	0.17
	PP (%)	4.75^a^ ± 0.13	4.61^b^ ± 0.13	4.61^b^ ± 0.14	0.0149	0.05
		AA (*n* = 92)	GA (*n* = 163)	GG (*n* = 89)		
g.304050 G>A	LL (d)	258.77 ± 7.58	258.30 ± 7.02	261.78 ± 7.06	0.3576	—
	PM (kg)	14.36 ± 0.03	14.49 ± 0.05	14.76 ± 0.05	0.9541	—
	MY (kg)	2750.84^b^ ± 174.28	2775.41^b^ ± 174.83	2859.77^a^ ± 172.96	0.0362	0.42
	FY (kg)	223.32 ± 8.92	221.05 ± 8.73	225.09 ± 8.04	0.5088	—
	FP (%)	8.11 ± 0.12	7.99 ± 0.11	7.94 ± 0.10	0.7526	—
	PY (kg)	127.57 ± 6.62	127.85 ± 6.92	131.37 ± 6.09	0.7746	—
	PP (%)	4.67^a^ ± 0.14	4.62^b^ ± 0.13	4.59^b^ ± 0.13	0.0060	0.16
		AA (*n* = 92)	GA (*n* = 167)	GG (*n* = 89)		
g.325647 G>A	LL (d)	258.77 ± 7.52	258.56 ± 7.83	261.78 ± 7.90	0.3547	—
	PM (kg)	14.36 ± 0.04	14.49 ± 0.04	14.76 ± 0.04	0.9677	—
	MY (kg)	2750.84^b^ ± 180.43	2777.19^ab^ ± 180.07	2859.77^a^ ± 181.07	0.0376	0.61
	FY (kg)	223.32 ± 6.73	220.88 ± 7.05	225.09 ± 7.09	0.4609	—
	FP (%)	8.11 ± 0.11	7.99 ± 0.12	7.94 ± 0.12	0.6956	—
	PY (kg)	127.57 ± 7.03	127.85 ± 7.82	131.37 ± 8.90	0.7688	—
	PP (%)	4.67^a^ ± 0.13	4.62^b^ ± 0.14	4.59^b^ ± 0.14	0.0056	0.37
		CC (*n* = 76)	TC (*n* = 119)	TT (*n* = 67)		
g.325817 T>C	LL (d)	258.09 ± 8.08	258.66 ± 7.90	261.14 ± 8.04	0.9884	—
	PM (kg)	14.56 ± 0.07	14.58 ± 0.08	14.73 ± 0.08	0.6488	—
	MY (kg)	2801.34 ± 176.90	2784.64 ± 179.52	2818.07 ± 180.11	0.6529	—
	FY (kg)	226.11 ± 7.82	221.14 ± 7.11	224.11 ± 7.49	0.5368	—
	FP (%)	8.06 ± 0.13	7.97 ± 0.12	7.97 ± 0.13	0.6884	—
	PY (kg)	129.81 ± 7.82	128.88 ± 7.11	129.87 ± 7.55	0.6718	—
	PP (%)	4.67 ± 0.11	4.64 ± 0.12	4.60 ± 0.13	0.8541	—
		AA (*n* = 92)	GA (*n* = 167)	GG (*n* = 89)		
g.325997 G>A	LL (d)	258.77 ± 8.90	258.56 ± 8.09	261.78 ± 8.11	0.3547	—
	PM (kg)	14.36 ± 0.06	14.49 ± 0.05	14.76 ± 0.06	0.9677	—
	MY (kg)	2750.84^b^ ± 180.11	2777.19^ab^ ± 181.17	2859.77^a^ ± 181.90	0.0376	0.28
	FY (kg)	223.32 ± 7.69	220.88 ± 7.92	225.09 ± 7.11	0.4609	—
	FP (%)	8.11 ± 0.14	7.99 ± 0.10	7.94 ± 0.11	0.6956	—
	PY (kg)	127.57 ± 6.90	127.85 ± 6.72	131.37 ± 6.11	0.7688	—
	PP (%)	4.67^a^ ± 0.10	4.62^b^ ± 0.10	4.59^b^ ± 0.10	0.0056	0.14

## Data Availability

The data used to support the findings of this study are included within the article.
